# Exploring consumers’ attitudes towards informal patient payments using the combined method of cluster and multinomial regression analysis - the case of Hungary

**DOI:** 10.1186/1472-6963-13-62

**Published:** 2013-02-15

**Authors:** Petra Baji, Milena Pavlova, László Gulácsi, Wim Groot

**Affiliations:** 1 Health Economics and Health Technology Assessment Research Centre, Corvinus University of Budapest, Fővám tér 8, Budapest, 1093, Hungary; 2 Center for Public Affairs Studies Foundation, Budapest, Hungary; 3 Department of Health Services Research; CAPHRI; Maastricht University Medical Center; Faculty of Health, Medicine and Life Sciences, Maastricht University, Duboisdomein 30, 6229 GT Maastricht., P.O. Box 616, Maastricht, MD, 6200, The Netherlands; 4 Topinstitute Evidence-Based Education Research (TIER), MaastrichtUniversity, Maastricht, The Netherlands

**Keywords:** Informal payments, Hungary, Consumer perceptions

## Abstract

**Background:**

Previous studies on informal patient payments have mostly focused on the magnitude and determinants of these payments while the attitudes of health care actors towards these payments are less well known. This study aims to reveal the attitudes of Hungarian health care consumers towards informal payments to provide a better understanding of this phenomenon.

**Methods:**

For the analysis, we use data from a survey carried out in 2010 in Hungary involving a representative sample of 1037 respondents. We use cluster analysis to identify the main attitude groups related to informal payments based on the respondents’ perception of and behavior related to informal payments. Multinomial logistic regression is applied to examine the differences between these groups in terms of socio-demographic characteristics, as well as past utilization and informal payments paid for health care services.

**Results:**

We identified three main different attitudes towards informal payments: accepting informal payments, doubting about informal payments and opposing informal payments. Those who accept informal payments (mostly young or elderly people, living in the capital) consider these payments as an expression of gratitude and perceive them as inevitable due to the low funding of the health care system. Those who doubt about informal payments (mostly respondents outside the capital, with higher education and higher household income) are not certain whether these payments are inevitable, perceive them as similar to corruption rather than gratitude, and would rather use private services to avoid these payments. We find that the opposition to informal payments (mostly among men from small households and low income households) can be explained by their lower ability and willingness to pay.

**Conclusions:**

A large share of Hungarian health care consumers has a rather positive attitude towards informal payments, perceiving them as “inevitable due to the low funding of the health care system”. From a policy point-of-view, the change of this consumer attitude will be essential to deal with these payments in addition to other policy strategies.

## Background

Informal payments for health care services present an important and challenging policy issue in most of the Central and Eastern European countries [1,2]. These payments violate the transparency in the financing of the health care systems and jeopardize the accountability of the providers. They also lead to inefficient use of health care resources [1-3] and inequalities in access to health care services [4-6].

The definition of informal payments varies across the literature and reflects cultural differences in the perception of informal payments [2,7,8]. However, authors agree that these payments are unofficial, i.e. they are outside the official payment channels (not registered by the state and made without an official receipt of payment). Various types of informal payments can be distinguished based on who initiates the payments (the patient or the provider), who receives the payment (medical staff, institution), who makes the payment (patient or relatives), what the nature of the payment is (cash or in-kind), when the payment is made (given ex-ante or ex-post), what the purpose/motivation of these payments is (patient's gratitude, tip or fee for service) [8]. Also the legal status of informal payments might differ across countries (whether it is explicitly forbidden by the law). They can be legal (not forbidden or even permitted by the law), or illegal (forbidden by law although sometimes condoned by governments) [8].

Overall, informal payments are seen as a rather complex phenomenon interrelated with different socio-cultural, legal-ethical and economic factors in a country. These factors have been extensively discussed in the literature [1,2,6,7,9-11]. According to the socio-cultural explanation, informal payments are considered as a tip and expression of patient’s gratitude (e.g. [4,7]). Based on the legal-ethical considerations, the existence of informal payments can be explained by the lack of control and accountability of governance structures [7]. The economic explanation mostly refers to the shortage of resources in the health care sector, low salaries of physicians and the existence of an informal market for services provided with better quality (e.g. [7,12,13]). However, the differentiation between these factors is rather difficult due to the “shadow” nature of the informal payments (i.e. the returns of informal payments cannot be measured and they are not compellable). Also, these factors might differ across countries. In low and middle income countries (e.g. Ukraine, Tajikistan, Armenia, Kyrgyzstan and Georgia), informal payments are an important source of health care financing [9,14]. While in Central Europe (in Hungary for example), informal payments mostly contribute to the salary of health care personnel [15].

Most of the empirical studies on informal patient payments aim to estimate the magnitude of these payments and their determinants, while there is less scientific evidence on the perception of health care actors related to these payments [8,16]. To be able to understand why informal payments are widespread, the perceptions and attitude of health care consumers towards these payments are one of the key factors (besides factors on the provider side). Thus, evidence on this issue may support policy making related to the eradication of informal payments.

In this study, we address the issue of informal payments, defined as unofficial cash or in-kind payments given to the health care personnel, in Hungary where such payments are widespread, especially in hospital care [6,16,17]. The aim of the study is to examine the perceptions of Hungarian health care consumers related to informal payments. For the analysis, we use data from a household survey carried out in 2010 in Hungary on a country-representative sample of 1037 respondents. We use cluster analysis to identify the main attitude groups related to informal payments. We also use multinomial logistic regression to examine the differences between these groups in terms of socio-demographic characteristics, as well as past utilization and informal payments for hospital services. Although we focus on Hungary, our results are relevant for other countries where informal payments are widespread.

### Background: informal payments in Hungary

In Hungary, some authors consider informal payments as the heritage of the socialist system while others argue that these payments existed even before the socialist period [18]. However, health care consumers are still regularly paying informally for health care services even 20 years after the fall of the communist regime. According to the results of a previous study, in 2007, 9% of the patients paid informally for their last visit to a GP (€2 on average), 14% paid informally for specialist care (€35 on average), and 50% paid informally for hospitalization (€58 on average) [17]. Informal payments are most widespread in case of gynecology visits, delivery and for surgical admissions [17,19].

The attitude of the Hungarian government towards informal payments has been rather controversial during the last decades. On the one hand, several Ministry Committees and policy measures have addressed the problem of informal payments in Hungary (including media campaigns against informal payments, the increase of the salaries in the public sector as well as the introduction of co-payments for health care services). However, despite these arrangements, there were no significant changes regarding the magnitude of these payments during this period [16,17]. On the other hand, the national regulations do not explicitly forbid informal patient payments. Since July 2012, the Labor Code in Hungary prohibits receiving informal payments. However, the employer has the right to dispense the employees from this decree. Thus, in a way, the government tolerates informal payments in the health care sector. The reason for this tolerance might be that informal payments contribute to the system funding by complementing the income of health care personnel [2,3,9].

This idea is supported by the standpoint of the medical profession, as the Ethic Codex of the Hungarian Medical Chamber declares that “…one of the explanations of the existence of informal payments is the low salary of the physicians and the dysfunction of the health care system.” ([20], quoted in [7]). The studies show that indeed, in Hungary, informal payments are paid to the medical staff and contribute to their salary [16,19]. According to the estimations of Gaál et al. (2006), physicians may have earned between 60% and 236% of their net official income from informal payments. However, they also point out that these payments are unequally distributed among physicians: 5% of them receive 60% of the informal payments [16]. This can imply that beneficiaries of informal payments might have the power to block important changes in the health care system to maintain the status quo.

Regarding the consumer side, the literature on Hungary suggests that informal payments are not only the expression of gratitude of health care consumers, but a kind of “fee for service” to receive better quality, quicker access and more attention [7]. More precisely, health care consumers pay informally because they are afraid that they will have less chance to obtain these services and benefits if they do not pay informally to the health care personnel [6,7,18]. Gaál and McKee (2004) consider informal payments as a reaction to the “declining” performance in the health care system [12]. According to the authors, dissatisfied health care consumers, who have no possibility to satisfy their needs elsewhere or cannot openly complain, are using informal channels (such as informal payments) to obtain the care they desire.

## Methods

### Data collection

The data used in the analysis, were collected in July 2010 in a household survey among adult citizens (age 18+), carried out as a part of an international research project. The objective of the survey was to collect data on past payments for health care services during the last 12 months, attitudes towards informal payments, preferences and willingness to pay for health care services. In Hungary, the survey was conducted via face-to-face individual interviews at the respondents’ home. The aim was to have 1000 effective interviews per country that present samples representative for the country.

The respondents were identified based on a multi-staged random probability method. During the first stage, sampling points in the country were selected. Within each of the seven regions in Hungary, the cities, towns and villages (rural area) included in the survey, were selected at random proportionally to regional and urban/rural characteristics of the population. In particular, the number of sampling points in the rural areas was calculated based on the ratio of the urban/rural population in the country. As the objective was to have 7–8 interviews per sampling point, in total, 132 sampling points (43 in rural area) have been included (36 in Central Hungary, 14 in Central Transdanubia, 13 in West Transdanubia, 15 in South Transdanubia, 16 in North Hungary, 19 in North Lowland and 17 in South Lowland). In the second stage, to select addresses/households of potential respondents, the random route method was used. For each sampling point, a starting point and direction were determined^a^. In the third stage, the selection of the respondent within the selected household was done using the “last birthday” principle. In this procedure, the interviewer asked to speak to the adult member of the household who had the last birthday. The last-birthday method is based on the assumption that the assignment of birthdates is a random process and also every household member has an equal chance of being selected (for further information see [21,22]).

As mentioned before, the aim was to have 1000 completed interviews (7–8 per sampling point). Thus, when the selection of a respondent at the third stage failed (including the cases when the household or the respondent with the last birthday in the household could not be successfully contacted after 3 attempts, or the respondent refused or was unavailable to take part in an interview), a replacing respondent was identified in the same sampling point following the second and third stage of the selection method (i.e. random route method to identify the household and the last birthday principle to identify the respondent within the household).

The interviews were carried out face-to-face by qualified and experienced interviews who attended a training prior to the survey to clarify the fieldwork standards and the specificities of the questionnaire. A high number of interviewers (130) were involved in the survey to avoid the interviewer bias that might occur when one interviewer carries out many interviews. Each interviewer carried out 6–8 interviews.

Altogether, 1376 respondents were successfully contacted^b^, out-of them 330 refused or were unable to participate in the survey. This resulted in a response rate (calculated as interviewed/successfully contacted respondents) of 76%. The final sample for Hungary contains data for 1037 respondents^c^, 104–285 interviews per region in 132 sampling points. After the data collection, about 10% of all respondents interviewed were re-contacted either by telephone or in person to verify that the interview had been carried out. The verification procedure did not indicate problems.

All respondents were asked for an informed consent at the start of the interview. The survey targeted the general public (not specific patient groups) and had the form of a consumer survey. Therefore, there was no need for an approval of an ethics committee (also there was no experiment on patients). The data collection was performed in accordance with the ICC/ESOMAR International Code of Marketing and Research^d^.

### The questionnaire

The questionnaire included questions about the respondents’ perception of informal payments as well as questions about the respondents’ perceived behavior related to informal payments. Informal payments were defined at the outset of the questionnaire as consisting of informal cash payments (such as gratitude cash payments or under-the-table cash payments) and gifts in kind for receiving medical services. It was specified in the questions that such payments can be given to physicians, medical staff or other personnel in health care facilities.

The exact wording of the questions used in our analysis is presented in Table 1 (a copy of the relevant parts of the questionnaire is provided in the Additional file 1). Questions in the first block (see Table 1) inquire whether the respondents agree with six different statements about informal payments. In particular, respondents were asked whether they consider informal payments as a form of corruption or as gratitude, whether they accept informal payments as inevitable because of the low health care funding, and whether they agree that these payments should be eradicated. The questions in the second block in Table 1 inquire the respondents whether five statements on behavior related to informal payments apply to them personally.

**Table 1:** Statements used in the cluster analysis

**Do you AGREE with the following statements? (yes/somewhat/no)**	
• Informal CASH payments to physicians and medical staff are similar to corruption.	
• Gifts IN KIND to physicians and medical staff are similar to corruption.	
• Informal CASH payments to physicians and medical staff are an expression of gratitude.	
• Gifts IN KIND to physicians and medical staff are an expression of gratitude.	
• Informal cash payments and gifts in kind to physicians and medical staff are INEVITABLE because of the low funding of the health care sector.	
• Cash or gifts in kind, given informally to physicians and medical staff, should be ERADICATED.	
**Do the following statements apply to YOU PERSONALLY? (yes/somewhat/no)**	
• I will feel UNCOMFORTABLE if I leave the physician's office without a gratitude cash payment or gift in kind.	
• I would RECOGNISE the hint of physicians or medical staff for an informal cash payment or a gift in kind.	
• I will REFUSE to pay if a physician or medical staff ask me to pay informally for a medical service.	
• I will PREFER to use private medical services if I have to pay informally for public medical services.	
• If I have SERIOUS PROBLEMS with my health, I will be ready to pay as much as I have in order to get better medical services.	

Other parts of the questionnaire included questions on the utilization and payments for physician services (including any physician in primary and outpatient specialist care both in the public and private sector excluding dentists) and hospitalization (including 1 day surgery as well as longer hospitalizations) during the preceding 12 months. Respondents were also asked about their attitude towards informal payments (cash payments and gifts in kind given to the health care personnel) and about socio-demographic characteristics.

### Analysis

We divide the respondents into attitude groups based on their individual responses. By applying the method of hierarchical cluster analysis with the Ward method, we explore the pattern of the respondents’ answers. The Ward method ensures homogeneity and relatively equal size of the groups. In a cluster analysis, the number of clusters is subjective. However, there are statistical tests, which can be used as a guidance to identify the “optimal” number of clusters. The two most frequently applied statistical tests - Duda-Hart test and Calanski Harabas test, provide us with a test on each stage of the clustering for deciding on whether or not the splitting of a cluster is justifiable [23,24]. In our analysis, both tests suggest 2 clusters as the best option (one group for and one group against informal payments). However, we decide to use 3 clusters, as we find it interesting to study the differences within the group of those who are against informal payments. We determined the final clusters using k-centered clustering with 3 groups (setting starting points as random 3 observations). Differences between the variables are calculated using the method of Euclidean-distance. We use ANOVA to test whether the clusters significantly differ from each other in terms of the variables used in the cluster analysis.

After the formation of the three groups, we apply multinomial logistic regression to examine the association between socio-demographic characteristics and cluster memberships. We estimate two models. In the first one, we use the cluster indicator (Group = 1,2,3) as a dependent variable and socio-demographic characteristics (such as age, gender, education of the respondent, logarithm of the monthly net household income, size of the household, place of residence of the household) as explanatory variables. In the second model, we also include past utilization and informal payments for physician or hospital services as explanatory variables. To examine the effect of past utilization and payments, we formulated three different groups for each service (i.e. visit to physician or hospitalization): those who did not use the service during the last 12 months; those who used the service but did not pay informally; those who used the service and paid informally. To define these groups, in the regression analysis, we include two indicators per service: (1) for not using a given service and (2) for using the services and paying informally. The categories that refer to using a given services and not paying informally are taken as reference categories.

## Results

Altogether, 924 persons gave valid answers to all questions in Table 1 and were classified into three groups (following the procedure described in the method section): Group 1 consists of 311 members, Group 2 with 316 members and group 3 with 297 members. Table 2 presents the characteristics of the three groups based on the variables used in the cluster analysis. According to the results of ANOVA analysis (Table 3) the groups significantly differ in terms of all variables used in the cluster analysis.

**Table 2:** Statements included in the cluster analysis presented by clusters

**Group**	**1**	**2**	**3**	**Total**
N	311	316	297	924
**I will feel UNCOMFORTABLE if I leave the physician's office without a gratitude cash payment or gift in kind.**	**1**	**2**	**3**	**Total**
No	170 (54.7%)	214 (67.7%)	252 (84.8%)	636 (68.8%)
Somewhat	72 (23.2%)	50 (15.8%)	30 (10.1%)	152 (16.5%)
Yes	69 (22.2%)	52 (16.5%)	15 (5.1%)	136 (14.7%)
**I would RECOGNISE the hint of physicians or medical staff for an informal cash payment or a gift in kind.**	**1**	**2**	**3**	**Total**
No	37 (11.9%)	30 (9.5%)	57 (19.2%)	124 (13.4%)
Somewhat	72 (23.2%)	67 (21.2%)	74 (24.9%)	213 (23.1%)
Yes	202 (65.0%)	219 (69.3%)	166 (55.9%)	587 (63.5%)
**I will REFUSE to pay if a physician or medical staff ask me to pay informally for a medical service.**	**1**	**2**	**3**	**Total**
No	134 (43.1%)	67 (21.2%)	49 (16.5%)	250 (27.1%)
Somewhat	85 (27.3%)	88 (27.8%)	51 (17.2%)	224 (24.2%)
Yes	92 (29.6%)	161 (50.9%)	197 (66.3%)	450 (48.7%)
**I will PREFER to use private medical services if I have to pay informally for public medical services.**	**1**	**2**	**3**	**Total**
No	171 (55.0%)	3 (0.9%)	212 (71.4%)	386 (41.8%)
Somewhat	76 (24.4%)	68 (21.5%)	70 (23.6%)	214 (23-2%)
Yes	64 (20.6%)	245 (77.5%)	15 (5.1%)	324 (35.1%)
**If I have SERIOUS PROBLEMS with my health, I will be ready to pay as much as I have in order to get better medical services.**	**1**	**2**	**3**	**Total**
No	27 (8.7%)	23 (7.3%)	119 (40.1%)	169 (18.3%)
Somewhat	67 (21.5%)	87 (27.5%)	123 (41.4%)	277 (30.3%)
Yes	217 (69.8%)	206 (65.2%)	55 (18.5%)	478 (51.7%)
**Informal CASH payments to physicians and medical staff are similar to corruption.**	**1**	**2**	**3**	**Total**
No	163 (52.4%)	7 (2.2%)	12 (4.0%)	182 (19.7%)
Somewhat	127 (40.8%)	105 (33.2%)	73 (24.6%)	305 (33.0%)
Yes	21 (6.8%)	204 (64.6%)	212 (71.4%)	437 (47.3%)
**Gifts IN KIND to physicians and medical staff are similar to corruption.**	**1**	**2**	**3**	**Total**
No	237 (76.2%)	79 (25.0%)	61 (20.5%)	377 (40.8%)
Somewhat	68 (21.9%)	112 (35.4%)	91 (30.6%)	271 (29.3%)
Yes	6 (1.9%)	125 (39.6%)	145 (48.8%)	276 (29.9%)
**Informal CASH payments to physicians and medical staff are an expression of gratitude.**	**1**	**2**	**3**	**Total**
No	17 (5.5%)	125 (39.6%)	157 (52.9%)	299 (32.4%)
Somewhat	86 (27.7%)	134 (42.4%)	117 (39.4%)	337 (36.5%)
Yes	208 (66.9%)	57 (18.0%)	23 (7.7%)	288 (31.2%)
**Gifts IN KIND to physicians and medical staff are an expression of gratitude.**	**1**	**2**	**3**	**Total**
No	7 (2.3%)	53 (16.8%)	95 (32.0%)	155 (16.8%)
Somewhat	47 (15.1%)	138 (43.7%)	141 (47.5%)	326 (35.3%)
Yes	257 (82.6%)	125 (39.6%)	61 (20.5%)	443 (47.9%)
**Informal cash payments and gifts in kind to physicians and medical staff are INEVITABLE because of the low funding of the health care sector.**	**1**	**2**	**3**	**Total**
No	51 (16.4%)	137 (43.4%)	180 (60.6%)	368 (39.8%)
Somewhat	117 (37.6%)	90 (28.5%)	80 (26.9%)	286 (31.0%)
Yes	144 (46.3%)	89 (28.2%)	37 (12.5%)	270 (29-2%)
**Cash or gifts in kind, given informally to physicians and medical staff, should be ERADICATED.**	**1**	**2**	**3**	**Total**
No	128 (41.2%)	15 (4.7%)	15 (5.1%)	158 (17.1%)
Somewhat	130 (41.8%)	79 (25.0%)	58 (19.5%)	267 (28.9%)
Yes	53 (17.0%	222 (70.3%)	224 (75.4%)	499 (54.0%)

**Table 3:** ANOVA test of the statements included in the cluster analysis

	**1**	**2**	**3**	**ANOVA**
**Statements**	**mean (sd)**	**mean (sd)**	**mean (sd)**	**F (p)**
**I will feel UNCOMFORTABLE if I leave the physician's office without a gratitude cash payment or gift in kind**	0.68 (0.82)	0.49 (0.76)	0.20 (0.51)	33.93 (0.000)
**I would RECOGNISE the hint of physicians or medical staff for an informal cash payment or a gift in kind.**	1.53 (0.70)	1.60 (0.66)	1.37 (0.79)	8.40 (0.000)
**I will REFUSE to pay if a physician or medical staff ask me to pay informally for a medical service.**	0.86 (0.84)	1.30 (0.80)	1.50 (0.76)	49.81 (0.000)
**I will PREFER to use private medical services if I have to pay informally for public medical services.**	0.66 (0.80)	1.77 (0.45)	0.34 (0.57)	449.50 (0.000)
**If I have SERIOUS PROBLEMS with my health, I will be ready to pay as much as I have in order to get better medical services.**	1.61 (0.64)	1.58 (0.62)	0.78 (0.74)	148.42 (0.000)
**Informal CASH payments to physicians and medical staff are similar to corruption.**	0.54 (0.62)	1.62 (0.53)	1.67 (0.55)	391.09 (0.000)
**Gifts IN KIND to physicians and medical staff are similar to corruption.**	0.26 (0.48)	1.15 (0.79)	1.28 (0.78)	195.03 (0.000)
**Informal CASH payments to physicians and medical staff are an expression of gratitude.**	1.61 (0.59)	0.78 (0.73)	0.55 (0.64)	224.37 (0.000)
**Gifts IN KIND to physicians and medical staff are an expression of gratitude.**	1.80 (0.45)	1.23 (0.72)	0.89 (0.72)	160.73 (0.000)
**Informal cash payments and gifts in kind to physicians and medical staff are INEVITABLE because of the low funding of the health care sector.**	1.30 (0.73)	0.85 (0.83)	0.52 (0.71)	80.67 (0.000)
**Cash or gifts in kind, given informally to physicians and medical staff, should be ERADICATED.**	0.76 (0.72)	1.66 (0.57)	1.70 (0.56)	226.10 (0.000)

### Results of the cluster analysis

Regarding the perceptions of informal payments, most of the respondents in Group 2 and Group 3 agree (65% and 71% respectively) or somewhat agree (33% and 25% respectively), that informal cash payments to physicians and medical staff are similar to corruption. Respondents in Group 1 either do not agree (52%) or only somewhat agree (41%) with this statement.

In Group 1, most of the respondents agree (67%) or somewhat agree (28%) that informal cash payments to physicians and medical staff are an expression of gratitude. Most of the respondents in Groups 2 and 3 somewhat agree (42% and 39%) or do not agree (40% and 53%) with this statement.

The results are similar regarding gifts in kind. However, in general, the shares of those who agree that gifts in kind given to physicians and medical staff are similar to corruption are lower in Group 1, 2 and 3 (2%, 40% and 50% respectively) compared to cash payments (7%, 65% and 71%). The shares of those who agree that gifts in kind given to physicians and medical staff are an expression of gratitude are higher in all groups (83%, 40% and 21%) compared to cash payments (67%, 18%, 8%).

Most of the respondents in Group 1 agree (46%) or somewhat agree (38%) that informal cash payments and gifts in kind to physicians and medical staff are inevitable because of the low funding of the health care sector. Group 2 is rather divided regarding this question, most of the respondents do not agree (43%), while 29% and 28% somewhat agree or agree with this statement. In Group 3, most of the respondents do not agree (61%) or somewhat agree (27%) with this statement.

In Group 1, most of the respondents somewhat agree (42%) or agree (41%) that cash or gifts in kind given informally to physicians and medical staff, should be eradicated. However, Group 2 and 3 are more likely to agree with this statement (in Group, 70% agree and 25% somewhat agree while in Group 3, these shares are 75% and 20%).

Regarding the respondents’ perceived behavior towards informal payments, respondents in Group 3 are less likely to feel uncomfortable when leaving the physician's office without a gratitude cash payment or gift in kind (85% would not feel uncomfortable) compared to Groups 1 and 2 (55% and 68% respectively). Groups 1 and 2 are more likely to feel uncomfortable in this situation (in Group 1 22% would feel uncomfortable, and 17% in Group 2) compared to Group 3, where only 5% would feel uncomfortable. Respondents in Group 3 are also less likely to recognize the hint of physicians or medical staff for an informal cash payment or a gift in kind (19% would not recognize such hint) compared to the Groups 1 and 2 (12% and 10% respectively). Respondents in Group 1 are less likely to refuse to pay if a physician or medical staff ask them to pay informally for a medical service (43% would not refuse to pay) compared to Groups 2 and 3 (21% and 17% respectively). Respondents in Group 2 are more likely to prefer to use private services to avoid paying informally (78% would use private services), while Group 3 is less likely to prefer to use private services (only 5% would use). This share is 21% in Group 2. Respondents in Group 3 are less likely to be ready to pay as much as they have in order to get better medical services in case of serious health problems, 40% would not be ready to pay as much as they have, compared to 9% and 7% in Groups 1 and 2 respectively.

Based on the above characteristics of the three groups, we define Group 1 as accepting informal payments, Group 2 as doubting about informal payments and Group 3 as opposing informal payments.

### Results of the multinomial logistic regression

Socio-demographic characteristics of the respondents are presented in Table 4 separately for the three attitude groups, i.e. for those who accept (Group 1), doubt (Group 2) and oppose (Group 3) informal payments respectively. The results of the multinomial logistic regression models are presented in Tables 5 and 6. In particular, Table 5 shows the predicted probability of belonging to each attitude groups. Table 6 presents the original and estimated cluster memberships. In the first model (where only socio-demographic features are included as independent variables), 43.5% of the cases are well predicted. In the extended model (where indicators of past utilization and past informal payments per services are also included as independent variables), 47.1% of the cases are predicted correctly.

**Table 4:** Socio-demographic characteristics by clusters

**Group**	**1**	**2**	**3**	**Total**	**Missing**
**N**	**311**	**316**	**297**	**924**	**113**
**Socio- demographic characteristics**					
**Age group**					
<35	104 (33.4%)*	100 (31.7%)	87 (29.3%)	291 (31.5%)	36 (31.9%)
35-59	119 (38.3%)**	148 (46.8%)	130 (43.8%)	397 (43.0%)	33 (29.2%)***
≥60	88 (21.9%)***	68 (18.0%)***	80 (26.9%)	236 (25.5%)	44 (38.9%)***
**Gender**					
Man	144 (46.3%)	140 (44.3%)**	151 (50.8%)	435(47.1%)	46 (40.7%)
Woman	167 (53.7%)	176 (55.7%)**	146 (49.2%)	489(52.9%)	67 (59.3%)***
**Residence**					
Village	71 (22.8%)***	101 (32.0%)	92 (31.0%)	264 (28.6%)	39 (34.5%)
Town	166 (53.4%)	182 (57.6%)***	149 (50.2%)	497 (53.8%)	55 (48.7%)
Capital	74 (23.8%)***	33 (10.4%)***	56 (18.9%)	163 (17.6%)	19 (16.8%)
**Education**					
Primary	64 (20.6%)	40 (12.7%)***	71 (23.9%)	175 (18.9%)	35 (31.0%)***
Secondary/vocational	197 (63.3%)	233 (73.7%)***	198 (66.6%)	628 (68.0%)	67 (59.3%)**
Tertiary	50 (16.1%)***	43 (13.6%)**	28 (9.4%)	121 (13.1%)	11 (9.7%)
**Health**					
Very bad. bad	43 (13.8%)	28 (8.9%)**	38 (12.8%)	109 (11.8%)	14 (12.4%)
Fair	82 (26.4%)	79 (25.0%)**	90 (30.3%)	251 (27.2%)	33 (29.2%)
Good	98 (31.5%)	134 (42.4%)***	88 (29.6%)	320 (34.6%)	35 (31.0%)
Very Good. excellent	88 (28.3%)	75 (23.7%)	81 (27.3%)	244 (26.4%)	31 (27.4%)
**Age**					
Mean	46.4	44.6***	47.0	46.0	49.4*
SD	(18.4)	(15.9)	(17.2)	(17.2)	(19.96)
**Income (HUF), 1 EUR ~ 285 HUF**					
Mean	164 727**	188 230***	152 787	168 697	157 021***
SD	(81 808)	(100 351)	(100 081)	(95 441)	(79 959)
N	301	299	292	892	105
**Household**					
Mean	2.6	2.9***	2.5	2.7	2.5
SD	(1.2)	(1.3)	(1.3)	(1.3)	(1.5)
Past utilization and payments					
**Physician visit**					
N	310	315	296	921	112
Not visited	60 (19.4%)	55 (17.5%)	61 (20.6%)	176 (19.1%)	31 (27.7%)**
Visited and not paid	158 (51.0%)***	211 (67.0%)**	214 (72.3%)	583 (63.3%)	69 (61.6%)
Visited and paid	92 (29.7%)***	49 (15.6%)***	21 (7.1%)	162 (17.6%)	12 (10.7%)*
**Hospitalization**					
N	311	315	297	923	113
Not hospitalized	228 (73.3%)***	258 (81.9%)	239 (80.5%)	725 (78.6%)	92 (81.4%)
hospitalized not paid	34 (10.9%)**	27 (8.6%)***	45 (15.2%)	106 (11.5%)	16 (14.2%)
hospitalized and paid	49 (15.8%)***	30 (9.5%)***	13 (4.4%)	92 (10.0%)	5 (4.4)**

stars indicate significant difference from the **3**^**rd **^**group** in terms of mean (t test), or proportion (z-test).

in the case of “missing” group stars indicate significant difference from the Total.

* p < 0,1; ** p < 0,05; *** p < 0,01.

**Table 5:** Results of multinomial logistic regression analysis

	**Model 1**			**Model 2**		
**VARIABLES**	**Pr(clust = 1)**	**Pr(clust = 2)**	**Pr(clust = 3**	**Pr(clust = 1)**	**Pr(clust = 2)**	**Pr(clust = 3)**
age < 35	0.0754*	−0.0367	−0.0387	0.0783*	−0.0287	−0.0495
	(1.889)	(−0.993)	(−1.030)	(1.909)	(−0.754)	(−1.319)
age ≥ 60	0.0848*	−0.0474	−0.0374	0.0636	−0.0409	−0.0227
	(1.920)	(−1.154)	(−0.923)	(1.391)	(−0.964)	(−0.548)
gender: woman	0.0173	0.0568*	−0.0741**	0.00993	0.0532	−0.0632*
	(0.525)	(1.750)	(−2.260)	(0.290)	(1.595)	(−1.899)
residence: capital	0.124***	−0.169***	0.0455	0.121**	−0.169***	0.0472
	(2.696)	(−4.487)	(0.999)	(2.539)	(−4.278)	(1.016)
residence: village	−0.0756**	0.0255	0.0500	−0.0866**	0.0241	0.0625
	(−2.019)	(0.678)	(1.294)	(−2.261)	(0.625)	(1.578)
education: primary	0.0578	−0.121***	0.0632	0.0528	−0.114***	0.0617
	(1.249)	(−2.963)	(1.399)	(1.097)	(−2.677)	(1.330)
education: tertiary	0.0964*	−0.0234	−0.0730	0.0974*	−0.0223	−0.0751
	(1.825)	(−0.483)	(−1.499)	(1.781)	(−0.449)	(−1.556)
health status: bad, very bad	0.0738	−0.0366	−0.0372	0.0472	−0.0299	−0.0172
	(1.362)	(−0.695)	(−0.767)	(0.836)	(−0.543)	(−0.331)
ln(income)	−0.00711	0.0826**	−0.0755**	−0.0306	0.0863***	−0.0556*
	(−0.229)	(2.561)	(−2.541)	(−0.962)	(2.599)	(−1.874)
number of household members	0.00688	0.0216	−0.0284*	0.0116	0.0240	−0.0356**
	(0.474)	(1.522)	(−1.955)	(0.773)	(1.636)	(−2.413)
not visited physician	-	-	-	0.0689	−0.0605	−0.00844
	-	-	-	(1.464)	(−1.436)	(−0.201)
visited physician and paid informally	-	-	-	0.271***	−0.0491	−0.222***
	-	-	-	(5.340)	(−1.053)	(−5.914)
not hospitalized	-	-	-	−0.00551	0.0881*	−0.0826
	-	-	-	(−0.103)	(1.721)	(−1.571)
hospitalized and paid informally	-	-	-	0.0818	0.0790	−0.161***
	-	-	-	(0.998)	(0.922)	(−2.680)
Pr	0,3426	0,3272	0,3303	0,3424	0,3376	0,3200
**Regression characteristics**	-		-	-	-	-
Observations	892	892	892	888	888	888
Pseudo R-squared	0.0395	0.0395	0.0395	0.0704	0.0704	0.0704
Chi2 test	77.34	77.34	77.34	137.3	137.3	137.3
p	0.000	0.000	0.000	0.000	0.000	0.000

Note: z-statistics in parentheses *** p < 0.01, ** p < 0.05, * p < 0.1.

**Table 6:** Original and predicted cluster membership

Model1*						
**Cluster/Predicted cluster**	**1**	**2**	**3**	**Total_predicted**	**Missing**	**Total**
1	107 (35,7%)	109 (36,3%)	85 (28,3%)	301	10	311
2	57 (19,2%)	171 (57,6%)	71 (23,9%)	299	17	316
3	76 (26,1%)	106 (36,4%)	110 (37,8%)	292	5	297
missing	30 (28,6%)	37 (35,2%)	38 (36,2%)	105	8	113
Model2** (extended model)						
**Cluster/predicted cluster**	**1**	**2**	**3**	**Total_predicted**	**Missing**	**Total**
1	112 (37,3%)	98 (32,7%)	90 (30,0%)	300	11	311
2	61 (20,5%)	152 (51,2%)	84 (28,3%)	297	19	316
3	49 (16,8%)	88 (30,2%)	154 (52,9%)	291	6	297
missing	26 (25,0%)	31 (29,8%)	47 (45,2%)	104	9	113

Note: *43,5% of the cases the predictid cluster is equal to the original cluster, **47,1% of the cases the predicted cluster is equal to the original cluster.

The probability of belonging to Group 1 (accepting informal patient payments) is significantly higher for those who are under the age of 35 (by 8% points), or above 60 (by 9% points). This probability is significantly higher also for those who are living in the capital (by 12% points), however, significantly lower for those who are living in a village (by 8% points). Tertiary education also increases the probability of belonging to this group by 10% points. The probability of belonging to Group 2 (doubting informal patient payments) is significantly higher for women (by 6% points), at the same time, significantly lower for those who are either living in the capital (by 17% points) or have finished primary education (by 12% points).The probability of belonging to Group 3 (opposing informal patient payments) is significantly higher for men (by 7% points), while each additional household member decreases the probability of belonging to this group by 3% points.

When we include the indicators of past utilization and past informal payments for health care services in the model, we observe that these variables explain group memberships very well. Those who visited a physician during the last 12 months and paid informally for this type of service, are more likely to belong to Group 1 that mostly accepts informal payments (the probability increases by 27% points), and are less likely to belong to Group 3 that mostly opposes informal payments (the probability decreases by 22% points). Those who paid informally for hospitalization during the last 12 months are also less likely to belong to Group 3 (the probability decreases by 16% points). However, those who were not hospitalized during the last 12 month, are more likely to belong to Group 2 that has doubts about informal payments (the probability increases by 9% points).

Socio-demographic characteristics of those respondents, who were not classified due to missing responses, are also presented in Table 4. They are significantly older, more frequently women, finished primary education, and have a significantly lower household income, compared to those who answered all questions presented in Table 1 and were included in the analysis. According to the predictions of the extended model (see Table 5) (including socio-demographic features, as well as past utilization and payments for health care services), 45% of those with missing responses would belong to Group 3 (opposing informal payments).

## Discussion

In this study, we have identified three different attitude groups of Hungarian health care consumers based on their opinion and perceived behavior concerning informal payments. This section discusses the main findings to obtain better insights in the consumers’ motivation to make informal payments for health care services.

### Interpretation of the results

We find Group 1 – involving either young people or elderly who predominantly live in the capital – to be the most tolerant towards informal payments as they mostly perceive these payments as inevitable due to the low funding of the health care system. The perception and attitude of this group corresponds with the “economic” explanation of informal payments, which refers to the shortage of resources in the health care sector, low salary of physicians and the existence of informal market for services with better quality [2,9,15].

Thus, one of the explanations for this attitude might be the perceived low quality of health care services in Hungary. Qualitative results show that health care consumers are not satisfied with the performance of the health care system, and associate the low quality with the lack of financial and human resources in the Hungarian health care system [25,26]. This perception might create a motivation for health care consumers to pay informally for health care services in order to obtain better care. According to the theory of Gaál and McKee (2004), who extend Hirschman’s theory of “Exit, Voice, Loyalty” on the informal payments phenomenon, informal payments can be interpreted as a reaction to the “declining” performance in the health care system. Dissatisfied health care consumers who have no possibility to satisfy their needs elsewhere (exiting the system is not possible) or cannot openly make complains (voice is not an option) are using informal channels, such as payment or connections to improve their situation, i.e. to assure better care [12]. The same idea is behind the theory of “Alternative Politics”, which refers to the “do-it-yourself” approach adopted by citizens to address their dissatisfaction with governmental services and find other means (e.g. informal payments) of satisfying their needs [27].

Another explanation for the tolerant attitude towards informal payments observed in Group 1 is the perceived low salary of the health care personnel, which according to the Group might legitimate the existence of informal payments. The definition of informal payments in the Ethic Codex of the Hungarian Medical Chamber – presented in the background section - suggests that informal payments keep physicians working in public health care institutions. Consequently, informal payments are an indication of the solidarity of health care consumers with the medical personnel. A similar finding was also reported in a previous study in Hungary, where 70% of the respondents agree or rather agree that the existence of informal payments indicate that physicians are “underpaid”. Also, 67% of the respondents in that study agreed or rather agreed that until physicians do not get proper salary they have the right to accept informal payments [19]. This may explain our finding that individuals in Group 1 consider informal payments as inevitable and that they feel rather uncomfortable when leaving the physician's office without gratitude cash payments or gifts in kind. They would also not refuse to pay informally if asked by the medical personnel.

Individuals in Group 1 do not consider informal payments as a corruption practice, consequently, they would not use private services either to avoid these payment. The explanation of this finding might be that the national legislation itself, do not explicitly forbid these payments (as mentioned in the background section). Furthermore, the Ethical Codex of the Hungarian Medical Chamber openly declares that these payments “within certain limits are legal and legitimate” [20].

Group 2 – with individuals mostly living outside of the capital, completed higher education, and having a higher household income, doubts informal payments. This group is divided regarding the statement that informal payments are inevitable because of the lack of resources in the health care system. However, they definitely consider informal cash payments as a corruption practice, and would rather use private services to avoid these payments. This attitude group rather supports the “legal-ethical explanation” of informal payments. According to this explanation, informal payments are rather the consequence of the lack of control and accountability of the governance [16], than of the lack of resources in the health care system. We also find that individuals in this group are both willing to pay for their health (they are the most ready to pay as much as they can in case of a serious health problem) and able to pay for health care services (since we find a significantly higher household income in this group compared to other groups). Thus, in their case, the extension of formal payment channels or the development of the private health care market might be an option to deal with informal payments.

Group 3 (mostly men from small households and low household income) is the least tolerant group towards informal payments and it is least likely to accept to pay informally for health care services as well. Individuals in this group consider informal payments as a corruption practice and do not agree that these payments are inevitable because of the low funding of the health care system. Similar to Group 2, the attitudes of this group correspond with the “legal-ethical explanation” of informal payments. However, our results suggest that this is not the only explanation for the attitude of this group. We find that although this group considers informal payments as a corruption practice, they would not use private services to avoid these payments. Besides, this is the group where the least respondents accept to pay for their health care in case of a serious health problem. These findings might suggest that they are less willing to pay for health care in general because either they object to pay for health care services in any form (formal or informal) or they are less able to pay for services (as we find a significantly lower household income among this group). Thus, they might be opposed to the increase in private financing in health care and the extension of formal payment channels. Since they are less likely to pay informally, they might also experience discrimination in the provision compared to those who accept informal payments and pay informally (Group 1 and 2).

### Comparison to previous studies

As we mentioned in the introduction, previous studies on informal payments have mostly focused on the magnitude of the payments. In a recent systematic literature review, Stepurko et al. (2010) [8] identify only six studies examining consumers or patients’ attitude towards informal payments in different countries (see [4,28-32])These studies are rather divided regarding their conclusions, namely the attitudes reported in these studies vary from strongly negative to tolerant, depending on the motivation of the payment. Another six studies examine the perception of informal payments. Three studies report that informal patient payments are perceived as tradition and gratuity [31,33,34], and two studies report that these payments are perceived as illegal behavior and corruption [35,36]. One study mentions both perceptions [4]. Similarly to our findings, the above-mentioned studies also report that consumers tolerate informal payments when these payments are initiated by the patient, or can be explained by the economic conditions (e.g. lack of resources, low salary of the physicians) (See e.g. [32,35]). In contrast to previous studies however, we use attitudes and perceptions to identify attitude groups that prevail in a country and to examine the characteristics of these groups. This enables us to better understand the motivations of different attitude groups in Hungary. The application of our analysis on data from other countries could show the cross-country relevance of our conclusions.

### Discussion of the limitations

In this section, we highlight some limitations of our study. The data collection was carried out via face-to-face interviews. This reduces non-responses, as the interviewer can guide the respondent [37]. However, informal payments are a sensitive topic. Therefore, when answering the questions on informal payments, some respondents could feel uncomfortable and would give socially desirable answers. Although we recognize that the collection of sensitive data via a self-administrated questionnaire has a higher potential to reduce this bias [38,39], we opted for a face-to-face interview in our survey to reduce the non-response. Altogether 113 respondents did not answer some questions and therefore were excluded from the cluster analysis. To address this issue we compared the socio-demographic characteristics of this group to those respondents who gave valid answers to identify significant differences between them.

Furthermore, we find that half of the respondents (51%) agree (or rather agree) that informal payments are an expression of gratitude, but at the same time consider these payments similar to corruption. This might suggest that respondents give inconsistent answers. However, the same phenomenon is found in a previous study as well [7]. Hungarian health care consumers often use the term gratitude to explain the motivation behind informal payments, even though in most of the cases they pay informally in order to receive a better quality care, quicker access or more attention. One explanation of this puzzle might be that in Hungary, the expression “gratitude” is used to denominate informal payments. Nevertheless, the Ethic Codex of the Hungarian Medical Chamber also defines these payments as an expression of gratitude.

## Conclusion and policy recommendations

In this study, we have identified the main attitudes toward informal patient payments among Hungarian health care consumers: accepting informal payments, doubting informal payments and opposing informal payments.

From a policy point of view, the attitude of the first group is the most challenging, as they considers informal payments as an expression of gratitude and at the same time inevitable because of the low funding of the health care system. This corresponds with the economic explanation of informal payments, which is also enhanced by the health care providers’ side in Hungary.

The behavior of the second group – those who doubt informal payments - is more promising from a policy point-of-view. They regard these payments as similar to corruption and would use private services to avoid these payments. Thus, in their case, the extension of formal payment channels or the private market of services might be an option to deal with informal payments, as they are both willing and able to pay for health care services.

We find that the reason behind the opposition towards informal payments observed in the third group is their lower acceptance and ability to pay for the services, rather than the “unofficial nature” of these payments. In their case, policy makers should focus on equity issues as they might also experience discrimination in the provision compared to those who accept informal payments and pay informally.

If the government wants to make a commitment to fight against informal payments, one of the essential steps should be the change of this positive attitude towards informal payments found in this study. Until health care consumers get the message both from the government and from the health care personnel, that these payments are acceptable and legitimate (moreover necessary), it cannot be expected that tolerant attitude towards informal payments will change. The tolerant attitude might change if these payments would be strongly discouraged by the government regulations. Also, they will be less acceptable for the public if it is highlighted that informal payments are not the solution to the wage problems for the majority of the health care personnel as only a small group of physicians benefit from these payments (these problems require other solutions). On the contrary, informal payments create adverse incentives for their beneficiaries, which can be against the general policy objectives, and they might have the power to block important changes in health care system to maintain the status-quo [2].

We recognize however, that the change in public attitudes will be only one step in policy strategies aiming at the eradication of informal patient payments. It should complement (rather than substitute) other important steps such as improving quality of and access to public health care services, as well as securing sufficient resources for the public health care sectors. Moreover, the issue of informal payments cannot be addressed as a separate problem of the health care system alone. Fundamental changes in and outside the health care system are necessary to achieve appreciable results regarding the eradication of these payments.

## Endnotes

^a^ The household selected for the survey, was every forth address on the left hand side of the street in urban areas, turning left at intersections and, after reaching a dead end, going back to the last crossing and further proceeding at random. In a block-of-flats of up to four floors, every fifth apartment household was selected, counting from the first apartment on the left of the ground floor. In cases of unsuitable household, the interviewers approached the apartment next-door and continued doing this until reaching a suitable household. At that point, the interviews resume the standard step of every fifth apartment. In a block-of-flats of 5 floors and more, the selection was every tenth apartment. In rural areas, every fourth inhabitable house on both sides of the interviewer’s route was selected. In compounds of several houses behind a common fence, the interviewer had to select the fourth one from the left (counting from the gate), or if there were less than four houses behind a common fence, then the interviewer went out of the common yard, counting the houses as if they were along the street.

^b^ In 178 cases the interviewer was not able to contact the household (e.g. nobody was at home after 3 visits, or the address was non-eligible - non-residential building, nobody lives at this address, empty dwelling/s, building) or was not able to contact the respondent who had the last birthday in the household (the respondent was not at home and the interviewer could not obtain appointment for a next visit).

^c^ Out of the 1046 interviews completed, in 9 cases, the interviews were discarded due to various deficiencies in data collected, such as over 40% non-answered questions or “don’t know” answers.

^d^ Deatails can be found at:

http://www.esomar.org/uploads/public/knowledge-and-standards/codes-and-guidelines/ESOMAR_ICC-ESOMAR_Code_English.pdf.

## Competing interests

The authors declare they have no competing interests.

## Authors’ contributions

PB have made substantial contributions to conception and design, carried out the analysis and drafted the manuscript. MP had contributions to the design, been involved in the analysis and helped to draft the manuscript. LG was involved in the formulation of the research question, in the introduction and background section as well as in the discussion of the results in Hungarian policy context. WG made substantial contributions to conception and design and critically revised the manuscript. All authors read and approved the final manuscript.

## Authors’ information

BP is a PhD researcher at Maastricht University, Teaching Assistant at the Health Economics and Health Technology Assessment Research at Corvinus University of Budapest, and junior researcher at Center for Public Studies Affaires Foundation. MP is Assistant Professor of Health Economics at Maastricht University, Department of Health Services Research; CAPHRI; Maastricht University Medical Center; Faculty of Health, Medicine and Life Sciences. GL is Professor at Corvinus University of Budapest, where he is the head of the Health Economics and Health Technology Assessment Research Centre. GL is also a senior researcher at Center for Public Studies Affaires Foundation. Wim Groot is Professor of Health Economics at Maastricht University, Department of Health Services Research; CAPHRI; Maastricht University Medical Center; Faculty of Health, Medicine and Life Sciences.

## Pre-publication history

The pre-publication history for this paper can be accessed here:

http://www.biomedcentral.com/1472-6963/13/62/prepub

## Supplementary Material

Additional file 1 Wording of the questionnaire. Click here for file
