# Supplementary material for: Exploring consumers’ attitudes towards informal patient payments using the combined method of cluster and multinomial regression analysis - the case of Hungary
Source: BMC Health Serv Res. 2013 Feb 15;13:62. doi: 10.1186/1472-6963-13-62 (PMC3606140; doi:10.1186/1472-6963-13-62)
Supplement: Additional file 1 — Wording of the questionnaire. [file 1472-6963-13-62-S1.pdf]

ASSPRO CEE 2007
Collaborative Focused Research Project
FP7-SSH-2007 Grant Agreement No.:

GALLUP International

LOGO
COMPANY

EUROPEAN COMMISSION
European Research Area
SEVENTH FRAMEWORK PROGRAMME
Funded under Socio-economic Sciences & Humanities

All notations in red indicate text that should be adjusted per country.

PROJECT: **ASSPRO CEE 2007**

SURVEY: **WILLINGNESS AND ABILITY OF PATIENTS TO PAY FOR MEDICAL SERVICES**

QUESTIONNAIRE - ROUND 1 / JUNE-JULY 2010 / COUNTRY

FIELDWORK CARRIED OUT BY: **<company name>**

FIELDWORK MANAGED BY: **BBSS Gallup International**

THE QUESTIONNAIRE SHOULD BE FILLED IN ONLY FOR RESPONDENTS WHO ARE 18 YEARS OLD OR OLDER.

FILL IN THE FOLLOWING INFORMATION:

**SAMPLING POINT**										
**REGION**										
**RESPONDENT ID NUMBER**										
**DATE OF THE INTERVIEW (DD/MM/YYYY)**			/			/	2	0	1	0
**START TIME (USE 24 HOURS CLOCK)**			:							
**INTERVIEWER ID NUMBER**										

© ASSPRO CEE 2007 / project no. 217431 / funded by the European Commission under FP7 Theme 8 SSH
No parts of this questionnaire may be used, translated, stored, published, copied or transmitted in any form and by any means(electronic, mechanical, copying, recording and etc.) without the written permission issued by the coordinators of project ASSPRO CEE 2007 (project no. 217431). For project details: www.assprocee2007.com

Partner in <country>: < Contact details project representative for the country>

Project coordinator: Dr. M.Pavlova; Scientific coordinators: Prof.Dr. Wim Groot and Prof.Dr.Frits van Merode
Department BEOZ; Faculty HMLS; Maastricht University; PO BOX 616, Maastricht 6200 MD, The Netherlands.
E-mail: M.Pavlova@BEOZ.unimaas.nl; Tel: +31-43-3881705

INFORMED CONSENT PROCEDURE

BEFORE YOU START, READ THE FOLLOWING TO THE RESPONDENT:

- The aim of this survey is to collect data on citizens' opinion about the quality, access and price of medical services they use.
- The survey is not commissioned by the government or a health insurer.
- This survey is part of an international research project funded by the European Commission. The same survey is carried out in several European countries.
- The data collected during the survey will be used for research purposes, namely for statistic analyses and reports.
- Your answers will not be related to your personal details (address, etc.) and will be completely confidential.
- Answers to all questions are highly important to the project, so we hope that you will share your opinions and thoughts by answering all questions in the questionnaire.

Do you agree to participate in this survey?		**1 = YES**<br>**0 = NO**

CONTINUE THE INTERVIEW ONLY IF THE RESPONDENT AGREES TO PARTICIPATE.

ASK THE QUESTIONS FOLLOWING THEIR ORDER IN THE QUESTIONNAIRE.

READ THE EXACT WORDING OF THE QUESTIONS, AND AFTERWARDS, IF NECESSARY, MAKE CLARIFICATIONS.

USE LOCAL CURRENCY FOR ALL RELEVANT QUESTIONS (XXX).

PLEASE TRY TO AVOID "DON'T KNOW" (DK) ANSWERS AND REFUSALS.
IF THE RESPONDENT REFUSES TO ANSWER, KEEP THE ANSWER BOX BLANK..

PART 1: USE AND PAYMENTS FOR PHYSICIAN SERVICES USED BY THE RESPONDENT

The first set of questions concerns medical services that YOU used during the last 12 months (June 2009 – May 2010), and the money that you paid out-of-pocket (or your family members paid on your behalf) for YOU receiving these services.

Out-of-pocket payments include OFFICIAL payments, for which one may usually receive a receipt or other document, INFORMAL cash payments (such as gratitude cash payments or under-the-table cash payments), or gifts in kind for receiving medical services.

Out-of-pocket payments EXCLUDE monthly payments for health insurance (or voluntary health accounts), as well as payments that the patient receives back from the state or a health insurer.

INSTRUCTION: QUESTIONS Q.1A /Q.3A DEFINE NEXT SECTIONS. PLEASE TRY TO AVOID "DON'T KNOW".

**Q.1A**	During the last 12 months, how many times did YOU PERSONALLY visit a physician or a physician visited you personally at your home, including any physician in both the public and private system? (Homeopaths and traditional healers who are not physicians, and also dentists are excluded.)	[ ] TIMES<br>0=None<br>99=DK<br>IF NONE, GO TO Q.2C
**Q.1B**	Considering all types of official and informal cash payments, and gifts in kind, how much IN TOTAL did you spend (out-of-pocket) on these visits EXCLUDING payments for travelling, transportation by ambulance and pharmaceuticals?	[ ] XXX<br>0=None<br>99=DK<br>IF NONE, GO TO Q.2A
**Q.1C**	How much of this amount approximately was for INFORMAL cash payments and gifts in kind?	[ ] XXX<br>0=None<br>99=DK

PART 2: USE AND PAYMENTS FOR HOSPITAL SERVICES USED BY THE RESPONDENT

**Q.3A**	During the last 12 months, how many times were YOU hospitalised (placed in a hospital), including day surgeries or day treatments? (Re-hospitalisation, i.e. repeated hospitalisation for the same health problem, should be counted separately as a different hospitalisation.)	[ ] TIMES<br>0=None<br>99=DK<br>IF NONE, GO TO Q.4C
**Q.3B**	Considering all types of official and informal cash payments, and gifts in kind, how much IN TOTAL did you spend (out-of-pocket) on these hospitalisations EXCLUDING payments for travelling, transportation by ambulance and pharmaceuticals?	[ ] XXX<br>0=None<br>99=DK<br>IF NONE, GO TO Q.4A
**Q.3C**	How much of this amount approximately was for INFORMAL cash payments and gifts in kind?	[ ] XXX<br>0=None<br>99=DK

© ASSPRO CEE 2007 / project no. 217431 / funded by the European Commission under FP7-SSH

PART 3: ATTITUDE TOWARDS INFORMAL PATIENT PAYMENTS

The following questions concern your attitude towards INFORMAL cash payments and gifts in kind to physicians, medical staff or other personnel in health care facilities. Your personal opinion is very important, no matter whether you used or not medical services. There are no wrong or right answers.

Do the following statements apply to YOU PERSONALLY?

**Q.8A**	I will feel UNCOMFORTABLE if I leave the physician's office without a gratitude cash payment or gift in kind.	☐	**2** = **YES**<br>**1** = **SOMEWHAT**<br>**0** = **NO**<br>**99=DK**
**Q.8B**	I would RECOGNISE the hint of physicians or medical staff for an informal cash payment or a gift in kind.	☐	**2** = **YES**<br>**1** = **SOMEWHAT**<br>**0** = **NO**<br>**99=DK**
**Q.8C**	I will REFUSE to pay if a physician or medical staff ask me to pay informally for a medical service.	☐	**2** = **YES**<br>**1** = **SOMEWHAT**<br>**0** = **NO**<br>**99=DK**
**Q.8D**	I will PREFER to use private medical services if I have to pay informally for public medical services.	☐	**2** = **YES**<br>**1** = **SOMEWHAT**<br>**0** = **NO**<br>**99=DK**
**Q.8E**	If I have SERIOUS PROBLEMS with my health, I will be ready to pay as much as I have in order to get better medical services.	☐	**2** = **YES**<br>**1** = **SOMEWHAT**<br>**0** = **NO**<br>**99=DK**

Do you AGREE with the following statements?

**Q.9A**	Informal CASH payments to physicians and medical staff are similar to corruption.	☐	**2** = **YES**<br>**1** = **SOMEWHAT**<br>**0** = **NO**<br>**99=DK**
**Q.9B**	Gifts IN KIND to physicians and medical staff are similar to corruption.	☐	**2** = **YES**<br>**1** = **SOMEWHAT**<br>**0** = **NO**<br>**99=DK**
**Q.9C**	Informal CASH payments to physicians and medical staff are an expression of gratitude.	☐	**2** = **YES**<br>**1** = **SOMEWHAT**<br>**0** = **NO**<br>**99=DK**
**Q.9D**	Gifts IN KIND to physicians and medical staff are an expression of gratitude.	☐	**2** = **YES**<br>**1** = **SOMEWHAT**<br>**0** = **NO**<br>**99=DK**
**Q.9E**	Informal cash payments and gifts in kind to physicians and medical staff are INEVITABLE because of the low funding of the health care sector.	☐	**2** = **YES**<br>**1** = **SOMEWHAT**<br>**0** = **NO**<br>**99=DK**

**Q.9F**	Cash or gifts in kind, given informally to physicians and medical staff, should be ERADICATED.	2 = YES<br>1 = SOMEWHAT<br>0 = NO<br>99=DK

PART 9: **SOCIO-DEMOGRAPHIC CHARACTERISTICS**

Next questions concern your social and demographic characteristics. The information that is required will not be related to your identity. The data are only necessary in order to analyse the results of this survey in a statistical way.

**Q.17A**	In which YEAR were you born?	1 9 _ _
**Q.17B**	**FILL IN THE RESPONDENT'S GENDER.**	1 = FEMALE<br>0 = MALE
**Q.17C**	**FILL IN THE TYPE OF RESPONDENT'S RESIDENCE PLACE.**	4 = THE CAPITAL<br>3 = CITY (MORE THAN 500,000 INHABITANTS)<br>2 = CITY (200,000 – 500,000 INHABITANTS)<br>1 = TOWN (UP TO 200,000 INHABITANTS)<br>0 = VILLAGE
**Q.17D**	**SHOW CARD 6.**<br><br>What is the level of your current EDUCATION or current study?	5= TERTIARY EDUCATION (ISCED 5+6)<br>4 = POST-SECONDARY NON-TERTIARY EDUCATION (ISCED 4)<br>3 = UPPER SECONDARY EDUCATION (ISCED 3)<br>2 = LOWER SECONDARY OR SECOND STAGE OF BASIC EDUCATION (ISCED 2)<br>1 = PRIMARY OR FIRST STAGE OF BASIC EDUCATION (ISCED 1)<br>0 = NOT COMPLETED PRIMARY EDUCATION (ISCED 0)
**Q.17E**	**SHOW CARD 7.**<br><br>What is your primary OCCUPATION (activities) at present?	8 = STUDENT (IN EDUCATION)<br>7 = EMPLOYEE (IN PAID JOB)<br>6 = OWN/FAMILY PRIVATE BUSINESS (INCLUDING SELF-EMPLOYED)<br>5 = UNEMPLOYED (JOB-SEEKING)<br>4= NOT EMPLOYED (NOT SEEKING FOR A JOB, INCL. HOUSEWIFE)<br>3 = PENSIONER (BECAUSE OF AGE)<br>2 = PENSIONER (BECAUSE OF ILLNESS)<br>1 = FARMER/AGRICULTURER<br>0 = SOLDIER

**Q.17F**	**SHOW CARD 8.**<br><br>What is your MARITAL status at present?	5 = NEVER MARRIED AND SINGLE<br>4 = LIVING WITH A PARTNER WITHOUT MARRIAGE<br>3 = MARRIED (LIVING TOGETHER)<br>2 = MARRIED (LIVING SEPARATELY)<br>1 = DIVORCED AND SINGLE AT PRESENT<br>0 = WIDOW/ER AND SINGLE AT PRESENT
**Q.18A**	**SHOW CARD 9.**<br><br>How do you perceive your present HEALTH status?	5 = PERFECT HEALTH<br>4 = VERY GOOD HEALTH<br>3 = GOOD HEALTH<br>2 = FAIR HEALTH<br>1 = BAD HEALTH<br>0 = VERY BAD HEALTH

<u>Part 10:</u> HOUSEHOLD CHARACTERISTICS AND HOUSEHOLD INCOME

The last set of questions concerns your household. Household is one person or a group of persons sharing a flat/house and having a common budget or common expenditure.

FAMILY MEMBERS LIVING TOGETHER ARE ONE HOUSEHOLD ONLY IF THEY ALSO HAVE A COMMON BUDGET OR COMMON EXPENDITURE. FAMILY MEMBERS WHO DO NOT LIVE TOGETHER ARE NOT ONE HOUSEHOLD.

**Q.20A**	How many PERSONS are there in your household (incl. you)?	PERSONS
**Q.20A**	**SHOW CARD 10.**<br><br>Please take a look at this card.<br>Could you tell me which of the following categories corresponds to the NET AVERAGE HOUSEHOLD INCOME per month (i.e. after tax income) – considering all household members and all sources - wages, social welfare, pensions, rents, fees, etc?	17 = R - MORE THAN 3000.- EURO<br>16 = Q - FROM 2001.- TO 3000.- EURO<br>15 = P - FROM 1501.- TO 2000.- EURO<br>14 = O - FROM 1001.- TO 1500.- EURO<br>13 = N - FROM 751.- TO 1000.- EURO<br>12 = M - FROM 601.- TO 750.- EURO<br>11 = L - FROM 501.- TO 600.- EURO<br>10 = K - FROM 451.- TO 500.- EURO<br>9 = J - FROM 401.- TO 450.- EURO<br>8 = I - FROM 351.- TO 400.- EURO<br>7 = H - FROM 301.- TO 350.- EURO<br>6 = G - FROM 251.- TO 300.- EURO<br>5 = F - FROM 201.- TO 250.- EURO<br>4 = E - FROM 151.- TO 200.- EURO<br>3 = D - FROM 101.- TO 150.- EURO<br>2 = C - FROM 76.- TO 100.- EURO<br>1 = B - FROM 50.- TO 75.- EURO<br>0 = A - LESS THAN 50.- EURO<br>99=DK

This is the end of the questionnaire.
Thank you for your participation!

END TIME
(USE 24 HOURS CLOCK) __ __ : __ __

© ASSPRO CEE 2007 / project no. 217431 / funded by the European Commission under FP7-SSH
